# Frequency and risk factors of musculoskeletal disorders among kitchen workers

**DOI:** 10.1186/s42506-023-00128-6

**Published:** 2023-02-20

**Authors:** Abeer Abdelsalam, Ghada O. Wassif, Waleed Salah Eldin, Mona A. Abdel-Hamid, Samia I. Damaty

**Affiliations:** Department of Community, Environmental & Occupational Medicine, Ain Shams University, Cairo, Egypt

**Keywords:** Work-related musculoskeletal disorders, Musculoskeletal disorders, Kitchen workers, Nordic musculoskeletal disorders questionnaire

## Abstract

**Background:**

Kitchen work is associated with exposure to heavy workload which has been linked to work-related musculoskeletal disorders (WRMSDs) and many other occupational hazards. This study aimed to measure the frequency of WRMSDs related to working in kitchens of two major Egyptian students’ hostels, to determine the associated risk factors, and determine the distribution of musculoskeletal problems in various joints in different job categories.

**Methods:**

A cross-sectional analytical study was carried out among 128 kitchen workers of two major students’ hostels in Cairo, Egypt. A structured interview questionnaire was used to collect information on personal and occupational data and the prevalence of musculoskeletal pain in the past 12 months based on the valid Nordic musculoskeletal disorders questionnaire (NMQ).

**Results:**

The study revealed that the majority of kitchen workers (90.6%) at the students’ hostel reported WRMSDs within the past 12 months. The lower back (64.8%), knee (46.9%), foot (46.1%), neck (29.7%), and shoulders (23.4%) were the most affected sites. Age, educational status, job category, job duration, and body mass index (BMI) were significantly associated with WRMSDs among kitchen workers.

**Conclusion:**

kitchen workers are at a high risk of musculoskeletal disorders (MSDs) due to the poor work environment, the massive workload, and great time pressure to perform their duties. Interventions such as hiring more workers of younger age, providing rest breaks, and health education about occupational hazards to reduce the risk of musculoskeletal problems should be reinforced.

## Introduction

Work-related musculoskeletal disorders (WRMSDs) are a common cause of occupational disability, injury, and lost time in various occupations [1–4]. WRMSDs are one of the world's most serious public health issues, causing poor quality of life, workplace injuries, and disability [5, 6]. As a result, it has been identified as one of the leading causes of human suffering, productivity loss, and societal economic burden [7, 8]. The World Health Organization (WHO) estimates that 50–70% of workers develop WRMSDs. About 1.71 billion people suffer from musculoskeletal disorders globally. Among musculoskeletal illnesses, lower back pain constitutes the highest burden with a prevalence of 568 million people [9].

Musculoskeletal disorders (MSD) can be discomfort, aches, pains, and inflammatory and degenerative diseases of the locomotor system. Treatment and recovery in more chronic cases are frequently unsatisfactory, with the potential for permanent disability and job loss [10].

In the food and beverage industries, WRMSDs are the leading causes of occupational illness. Poor work organization and a heavy manual workload are the most common causes [11].

Cooks and food service workers (CFSWs) are important occupational groups in a variety of institutions, including hotels, prisons, hospitals, and universities. Because of the nature of their work and the types of raw and finished materials they handle, this group is expected to have a high incidence of occupational injuries and diseases [12].

Preparation of food for a considerable number of students within a predetermined time causes a huge workload and pressure on the kitchen workers. As a result, kitchen work includes physical, mechanical, and psychosocial hazards. These workers are under a lot of stress and have a lot of work to do because there is not enough manpower and they are under a lot of mental and physical strain to keep the food quality [13].

Kitchen work leads to a high rate of musculoskeletal complaints due to the activities that require heavy lifting and a prolonged awkward posture. Long periods of standing, walking, highly repetitive motions, and forceful exertion are all linked to musculoskeletal problems among kitchen workers [14].

There is a scarcity of research involving prevalence and risk factors of MSDs among workers in the hospitality industry particularly in the kitchen in Egypt and the Arab region. Many studies had been conducted outside the Arab region; however, these studies involved kitchens in restaurants serving small populations. The present study was carried out in two kitchens located in two major students’ hostels in Cairo serving meals for more than 4000 students each day. The aim of the study was to measure the frequency of WRMSDs related to working in kitchens of a major Egyptian students’ hostel; to determine the risk factors associated with the occurrence of WRMSDs; and to determine the distribution of musculoskeletal problems in various joints in different job categories.

## Methods

This cross-sectional analytical study was conducted over one year from April 2021 to April 2022 among kitchen workers from two hostels serving students at a major Egyptian university in Cairo. It offers more than 4000 meals per day mainly for the students in male and female students’ hostels. Also, it provides meals to a number of employees at the studied university (200-300 meals per day).

### Study Subjects

All kitchen workers (*n*=128) in the students’ hostels were included and responded to the questionnaire. The present study group consists of chief cooks (*n*=23), assistant cooks (*n*=22), laborers (*n*=25), food presenters (*n*=15), supervisors (*n*=22), administrative employees (*n*=17), and maintenance workers (*n*=4). All are full-time workers performing their duties for at least 6 h/day, 5 or 6 days per week.

### Study Tools

Data were collected with an interview questionnaire which consisted of three sections. In the first section, sociodemographic characteristics of the study participants were recorded such as age, gender, residence, education, marital status, and smoking behavior. The second section of the questionnaire consisted of job characteristics such as job category, duration of working hours per day, and total work experience. The third section of the questionnaire was the Nordic Musculoskeletal questionnaire [15] which provided information about MSD among participating workers, including frequency (in the last week and in the last 12 months) and severity of pain which might lead to disabling MSDs. It also deals with the distribution of these disorders by body organ. Akrouf et al. (2010) [16] translated the Nordic questionnaire into an Arabic version and then retranslated it back into an English version to ensure correct translation. The researchers also assessed the questionnaire through a jury of 6 occupational and psychiatrist consultants to ensure its validity. The Cronbach reliability coefficient was more than 0.87 for the questionnaire.

Anthropometric measurements such as weight and height were done on the participating workers. Body mass index was calculated using the following formula: BMI = weight (kg)/height^2^ (m^2^).

### Statistical analysis

Data were statistically analyzed using SPSS version 23.0. Chi-square test and independent sample *t*-test were used to assess the relationship between sociodemographic and workplace factors (independent variables) and reported musculoskeletal complaints (dependent variable). *P*≤0.05 was considered statistically significant. Logistic regression analysis was used to determine the significant predictors of WRMSDs in the participating kitchen workers.

## Results

Concerning the sociodemographic characteristics of the participating kitchen workers; about half of the study population (49.2%) were between 40 and 50 years. The least frequent age group was the less than 30 years group (3.2%), about two-thirds (66.4%) were males, living in Cairo (63.3%), The majority (93%) were married, 45.3% and 31.3% of the study participants finished their primary/preparatory and secondary education, respectively. Regarding their smoking status, 41.4% were current smokers, and about half of them (51.1%) smoke 1–2 packs per day. The average smoking duration (years) was 22 ± 9.2. More than one-third (36.7%) were overweight, and 28.9% and 10.2% were classified as obese grade 1 and obese grade 2, respectively (Table 1).

**Table 1:** Sociodemographic characteristics of the participating kitchen workers in the studied student hostels, Cairo, Egypt (*n*=128)

Variable	*N*	%
**Age**		
<30	4	3.2
30–40	35	27.3
40–50	63	49.2
>50	26	20.3
Mean ± SD	44.8 ± 8.24	
Range	(24–59)	
**Gender**		
Male	85	66.4
Female	43	33.6
**Residence**		
Cairo	81	63.3
Outside Cairo	47	36.7
**Marital status**		
Single/widowed	9	7.0
Married	119	93.0
**Education**		
Illiterate	16	12.5
Primary/preparatory	58	45.3
Secondary	40	31.3
University or postgraduate studies	14	11.0
**Smoking status**		
Non-smoker	65	50.8
Current smoker	53	41.4
Past smoker	10	7.8
**Smoking type** (*n*=50)		
Cigarette	48	90.6
Shisha	5	9.4
**Smoking frequency** (cigarettes) (*n*=45)		
<1 pack	18	38.3
1–2 packs	24	51.1
>2 packs	5	10.6
**No. of cigarettes**		
Mean ± SD	19 ± 12	
Range	(2–60)	
**Smoking duration** (years)		
Mean ± SD	22 ± 9.2	
Range	(2–40)	
**BMI** (kg/m^2^)		
Normal (18–25)	30	23.4
Overweight (25–<30)	47	36.7
Obese 1 (30–<35)	37	28.9
Obese 2 (35–<40)	13	10.2
Obese 3 (more than 40)	1	0.8
Mean ± SD	28.9 ± 4.7	
Range	(18.83–40.12)	

Regarding job characteristics of the participating kitchen workers, 18% of the study participants were cooks, 17.2% were cooking assistants, 19.5% were laborers, 11.7% were food presenters, 13.3% were administrative employees, 17.2% were supervisors, and 3.1% were maintenance workers. The majority of the participating kitchen workers (84.4%) had been working in their current job for more than 10 years; the average job duration was 18.0 ± 9.0 years. All worked < 8 h per day and < 48 h per week. The majority (88.3%) worked 5 days a week. None of the study participants were periodically vaccinated with the routine vaccines for food handlers, nor performed regular lab tests of food handlers (Table 2).

**Table 2:** Job characteristics of the participating kitchen workers (*n*=128) in the studied student hostels, Cairo, Egypt (*n*=128)

	*N*	%
**Job**		
Cook	23	18.0
Assistant Cooker	22	17.2
Laborer	25	19.5
Food presenter	15	11.7
Adm employee^a^	17	13.3
Supervisor	22	17.2
Maintenance workers	4	3.1
**Job duration** (years)		
<5 years	15	11.7
5–10 years	5	3.9
>10 years	108	84.4
Mean ± SD	18 ± 9	
Range	(1–40)	
**Working hours per day**		
≤8 h	128	100.0
Mean ± SD	6.2 ± 0.64	
Range	(6–8)	
**Working hours per week**^b^		
≤48 h/week	128	100.0
Mean ± SD	32 ± 5.7	
Range	(30–48)	
**Working days**		
5 days	113	88.3
6 days	15	11.7
Mean ± SD	5.1±0.32	
Range	(5–6)	
**Vaccination**^c^		
Yes	0	0.0
No	128	100.0
**Periodic lab tests**^d^		
Yes	0	0.0
No	128	100.0

^a^Administrative employees include nutrition specialists, Employees, Veterinarians, and Store worker

^b^Work hours per week are classified into two categories: ≤48 h/week or >48 h/week guided by the Egyptian Labor Law which specifies that maximum working hours per day are 8 h or 48 h per week in case of a 6-day workweek

^c^Vaccination for Salmonella, hepatitis A virus, hepatitis B virus, and tetanus

^d^Periodic lab tests such as urine, sputum, and stool samples

Distribution of the participating kitchen workers according to the Standardized Nordic Questionnaire showed that the lower back (53.1%), knee (40.6%), foot/ankle (38.3%), and neck (24.2%) were the most affected parts during the previous week. The same percentages also apply for the affected body sites during the previous 12 months as follows; lower back (64.8%), knee (46.9%), foot (46.1%), neck (29.7%), and shoulders (23.4%). The most disabling MSDs affecting study subjects were the lower back (33.6%), knee (30.5%), foot (21.1%), and shoulder (15.6%) (Table 3).

**Table 3:** Distribution of the participating kitchen workers according to the Standardized Nordic Questionnaire (*n*=128)

Body part	Previous 7 days		Previous 12 month		Disabling attack	
	*N*	%	*N*	%	*N*	%
Neck	31	24.2	38	29.7	17	13.3
Shoulder	28	21.9	30	23.4	20	15.6
Upper back	22	17.2	26	20.3	11	8.6
Elbow	12	9.4	15	11.7	9	7.0
Wrist/hand	22	17.2	24	18.8	19	14.8
Lower back	68	53.1	83	64.8	43	33.6
Hip	8	6.3	8	6.3	5	3.9
Knee	52	40.6	60	46.9	39	30.5
Foot/ankle	49	38.3	59	46.1	27	21.1

 A statistically significant relationship between the occurrence of MSDs in the previous 1 year and age, education, job category, job duration, and BMI among there studied kitchen workers (*P*≤0.05), where work-related MSDs in the previous 1 year were more common among participants with older age, lower education, non-administrative job categories, longer job duration, and higher BMI (Table 4).

**Table 4:** Risk factors for MSDs among participating kitchen workers (*n*=128)

	No MSDS *N* (%)	MSDS *N* (%)	*P*-value^#^
**Age**			
20–30	2 (50)	2 (50)	0.007**
31–40	6 (17.1)	29 (82.9)	
41–50	4 (6.3)	59 (93.7)	
51–65	0 (0.0)	26 (100)	
**Gender**			
Male	8 (9.4)	77 (90.6)	0.984
Female	4 (9.3)	39 (90.7)	
**Residence**			
Cairo	9 (11.1)	72 (88.9)	0.376
Outside Cairo	3 (6.4)	44 (93.6)	
**Marital status**			
Single or widow	1 (11.1)	8 (88.9)	0.853
Married	11 (9.2)	108 (90.8)	
**Education**			
Illiterate	0 (0.0)	16 (100)	0.021*
Primary /preparatory	3 (5.2)	55 (94.8)	
Secondary	3 (7.5)	37 (92.5)	
University/postgraduate studies	5 (38.5)	8 (61.5)	
**Smoking**			
Non-smoker	9 (13.8)	56 (86.2)	0.270
Smoker	3 (5.7)	50 (94.3)	
Past smoker	0 (0.0)	10 (100.0)	
**Job**			
Cooker	2 (8.7)	21 (91.3)	0.002**
Cooking assistant	2 (9.1)	20 (90.9)	
Laborer	1 (4.0)	24 (96.0)	
Food presenter	0 (0.0)	15 (100.0)	
Adm. Employee	7 (41.2)	10 (58.8)	
Supervisor	0 (0.0)	22 (100.0)	
Maintenance worker	0 (0.0)	4 (100.0)	
	**Mean±SD**	**Mean±SD**	***P*-value**^**$**^
**Job duration**	12±7.27	19.12±8.97	0.01*
**BMI**	26.03±3.95	29.03±4.7	0.035*

^**#**^ Chi-square test was used but if (20.0%) of the cells or more have an expected count of less than 5 Fischer’s exact test was used

^**$**^ Independent sample ***t*** test was used

^*^ Statistically significant at ***P***≤0.05

^**^ Statistically significant at ***P***≤0.01

Distribution of musculoskeletal problems in various joints in different job categories (as per Standardized Nordic Questionnaire) showed that cooking assistants are the most exposed among all kitchen workers to most MSDs as neck pain, shoulder pain, elbow pain, wrist/hand pain, knee, and foot pain, while Laborers were most exposed to lower back pain. A statistically significant difference was found between different job categories regarding the occurrence of the elbow, low back, wrist/hand, and knee pain (*P*≤0.05) (Table 5).

**Table 5:** Musculoskeletal problems in various joints in different job categories (as per Standardized Nordic Questionnaire)

Variable	Chief cook (*n*=21) *N* (%)	Cooking assistant (*n*=20) *N* (%)	Laborer (*n*=24) *N* (%)	Food presenter (*n*=15) *N* (%)	Adm employee (*n*=10) *N* (%)	Supervisor (*n*=22) *N* (%)	Maintenance (*n*=4) *N* (%)	*P*-value^#^
Neck	4 (19.0)	11 (55.0)	7 (29.1)	5 (33.3)	4 (40.0)	6 (27.3)	1 (25.0)	0.364
Shoulder	7 (33.3)	8 (40.0)	6 (25.0)	5 (33.3)	1 (10.0)	2 (9.1)	1 (25.0)	0.132
Upper back	5 (23.8)	5 (25.0)	6 (25.0)	2 (13.3)	2 (20.0)	6 (27.3)	0 (0.0)	0.868
Elbow	0 (0.0)	7 (35.0)	5 (20.8)	1 (6.7)	1 (10.0)	1 (4.5)	0 (0.0)	0.018*
Lower back	13 (61.9)	17 (85.0)	21 (87.5)	10 (66.7)	6 (60.0)	14 (63.6)	2 (1.6)	0.041*
Wrist/hand	2 (9.5)	8 (40.0)	9 (37.5)	2 (13.3)	1 (10.0)	1 (4.5)	1 (25.0)	0.012*
Hip	1 (4.7)	4 (20.0)	1 (4.1)	0 (0.0)	0 (0.0)	1 (4.5)	1 (25.0)	0.124
Knee	10 (47.6)	16 (80.0)	13 (54.1)	6 (40)	4 (40.0)	8 (36.0)	3 (75.0)	0.05*
Foot	9 (42.8)	14 (70.0)	14 (58.3)	8 (53.3)	4 (40.0)	9 (40)	1 (25.0)	0.178

^**#**^ Chi-square test was used but if (20.0%) of the cells or more have an expected count of less than 5 Fischer’s exact test was used

^*^ Statistically significant at ***P***≤0.05

Logistic regression analysis revealed that job categories and education level were significant predictors of WRMSDs in the participating kitchen workers (Table 6).

**Table 6:** Logistic regression analysis revealing significant predictors of WRMSDs in the participating kitchen workers

Variable	***B***		Wald	Sig.	Exp(B)	95% CI for EXP(B)	
						Lower	Upper
**Age**	0.052	0.073	0.510	0.475	1.054	0.913	1.216
**Gender**	1.125	1.227	0.840	0.359	3.079	0.278	34.104
**Residence**	0.502	0.991	0.257	0.612	1.652	0.237	11.520
**Marital status**	0.392	1.996	0.039	0.844	1.480	0.030	74.060
**Education**	-1.061	0.376	7.958	0.005*	0.346	0.166	0.723
**Smoking status**	1.216	0.903	1.813	0.178	3.373	0.575	19.801
**Job categories**	0.660	0.337	3.839	0.050*	1.936	1.000	3.748
**Job duration**	0.020	0.076	0.068	0.794	1.020	0.879	1.184
**BMI**	0.127	0.098	1.681	0.195	1.135	0.937	1.375
**Constant**	−5.214	5.525	0.891	0.345	0.005		

^*^ Statistically significant at ***P***≤0.05

## Discussion

Kitchen work is a high-risk occupation for WRMSDs due to poor work environment, the intensive manual workload, and repetitive movements that are involved. The present study revealed that the majority of kitchen workers (90.6%) at the students’ hostel reported WRMSDs within the past 12 months.

The high prevalence of WRMSDs in the present study was similar to many studies conducted among kitchen and restaurant workers in Finland [17] (87%), Taiwan [18] (85.2%), Ethiopia [19] (81.5%), and Bangladesh [20] (78%). In contrast, the present finding was higher than studies conducted among kitchen workers in Spain [21] (69.2%), India [22] (67.5%), and Turkey [23] (59%).

The first possible reason of this contradiction is that kitchen workers in the present study are exposed to a more extensive manual working process as they are used to work under pressure serving a large number of meals every day at the students’ hostels which would induce higher degrees of exhaustion. The second reason could be the presence of poor occupational health and safety services in Egypt compared to other countries due to inadequate health and safety experts which would lead to poor and unsafe work environment, inadequate use of personal protective equipment (PPE), and poor knowledge among kitchen workers. A third possible explanation could be a variation in the assessment tools. A self-reported interview questionnaire was used in the present study. However, different study tools such as an ergonomic measurement tool was used in the Indian study [22] for instance. The fourth explanation might be due to the inclusion of kitchen workers with mean age (44.8 ± 8.24) which is higher than other studies as for instance the study carried out in Taiwan [18], where the mean age among cookers was 33.3 ± 11.3 years. In the current study most of the study participants were older than 30 years with the nearly half of the participants (49.2%) between 40 and 50 years. The older age of kitchen workers is attributed to the hiring freeze of new workers in the governmental sector in Egypt as it is saturated with the current employees who are growing older nowadays and are exposed to a higher prevalence of WRMSDs. As workers grow older, they are exposed to the diminution of bone density which begins at the age of 30 years. Low back and knee pain subsequently become more common.

The present study reported that the lower back (64.8%), knee (46.9%), foot (46.1%), neck (29.7%), and shoulders (23.4%) were the most affected parts during the previous 12 months.

The presence of low back pain as the chief complaint for kitchen workers was also reported in similar studies conducted in India [22] (65.8%), Ethiopia [19] (53.55%), Finland [17] (50%), and Malaysia [14] (43.3%), but higher than the studies conducted in India [24] (43%), Bangladesh [20] (38%), and Taiwan [18] (32.7%). The possible reason might be attributed to different participants’ characteristics and variations in workload. The percentage of low back pain in kitchen workers was higher than other occupations that did not require manual handling as computer workers [24] where the prevalence rate of lower back pain was reported as 40.4%, Also, the percentage was higher than a sample of Egyptian female hairdressers where the prevalence rate of lower back pain was 12.5% [25]

In addition to that, similar percentages for other body parts affected with MSDs was reported in a study done among restaurant workers in Ethiopia [19] where the percentages of discomfort or pain in the neck was (36.1%), shoulder (44.7%), hips/tights (33.6%), knee (40.7%), and ankle or foot (41.3%). In India [22] the shoulder region (62.3%), knee/foot (42.1%), neck region (38.6%), elbow/forearm (31.6%), and thigh (30.7%) were also among the most common affected body parts. This was higher than studies done in Taiwan [18], Bangladesh [20], Spain [21], and Turkey [23] among restaurant workers and the study done among hairdressers in Egypt [25]. Kitchen workers are exposed to MSDs in many body parts as compared to other occupations as restaurant workers’ tasks necessitate repetitive and forceful motion of the hand, raised shoulders, and bending of the back; these working postures induce pain in both lower and upper body parts. In addition to that, restaurant workers perform tasks in a static standing position for long duration leading to pain in the leg muscles.

Concerning risk factors of MSD in the studied kitchen workers; the present study revealed that age, educational status, job category, job duration and BMI were significantly associated with WR MSDs among kitchen workers.

Concerning the relationship between age and WRMSDs, the present study found that the prevalence of WRMSDs significantly increases as the participants’ age increase. This finding is supported by studies done by in Ethiopia [19], Bangladesh [20], South India [22], South Korea [26], and Iran [27]. The possible clarification is that biological structures of the human body, particularly related to muscles, joints, nerves, ligaments, and tendons degenerate as the ages of the workers increase in addition to diminution of bone which would provoke pain and result in higher tendencies of WRMSDs development.

The present study found that restaurant workers who were illiterate or attended primary and preparatory education were at higher odds of developing WRMSDs compared with those at a higher educational level. Manual work is usually assigned to low educated persons. Furthermore, education level was negative predictor for occurrence of work-related musculoskeletal disorders as education level improve the human knowledge about health. This is similar to a study conducted in Ethiopia [19], where the odds of developing WRMSDs among respondents who were illiterate or completed the primary levels of education were 1.79 and 2.14 times, respectively, than that of those who completed higher education. This finding is supported by a study conducted in Egypt [28] which showed that improving workers’ knowledge and practice regarding work-related musculoskeletal disorders could significantly minimize the burden of MSDs among Kitchen workers.

A statistically significant relationship was found between job category and WRMSDs, where cooks, cooking assistants, laborers and food presenters experienced MSDs at a higher percentage in the previous 12 month. This could be explained the heavy workload they had to perform. Their job necessities heavy lifting, repetitive monotonous work, awkward position, and long working time standing. In addition, there is a shortage in the number of the before mentioned job categories and their ages is mostly above forty. Also, job categories were positive predictors for occurrence of work-related musculoskeletal disorders. This was consistent with the results obtained from a study done in India [22] among kitchen workers; where chief cooks (79.2%) and assistant cooks (74.3%) reported highest MSDs.

This study revealed that job duration is an important work-related risk factor for musculoskeletal complaints. This is consistent with the results from studies done by in Kuwait [16], Taiwan [18], and Iran [27].

The current study also reported that higher BMI was associated with incidence of WRMSDs. This was similar to the studies done in Ethiopia [19], Taiwan [18, 29], Finland [30], Iran [27], Norway [31], and Canada [32]. In the study done in Iran [27], workers with higher BMI experienced a significantly higher rate musculoskeletal problems (*P*<0.001). Also, in Ethiopia [19] the odds of developing WRMSDs among kitchen workers with higher BMI were 1.14 than those with lower BMI. Participants in the study done in Taiwan [29] with BMI>27 kg/m2 were more likely to suffer from MSDs.

### Limitations

One of the limitations of this study was the possibility for recall bias due to the reliance on a self-reported questionnaire to collect data. However, we believe that this limitation has insignificant impact on our findings as the Nordic Musculoskeletal Questionnaire (NMQ) has been proven to have adequate reliability and validity. Furthermore, the sample was heterogeneous (It included chefs, cooking assistants, laborers, supervisors, administrative and maintenance workers with different pattern of work which could affect both occurrence and location of pain or discomfort).

## Conclusion

Kitchen workers are at a high risk of musculoskeletal disorders. Age, educational status, job category, job duration and BMI were significantly associated with WRMSDs among kitchen workers. Interventions as hiring adequate number of workers with younger age, providing rest breaks, and health education about occupational hazards to reduce the risk of work-related musculoskeletal problems should be reinforced. In addition, prospective study is needed to confirm the relationships showed by the current cross-sectional study.

## Data Availability

The data that support the findings of this study are available from the corresponding author upon request.

## Abbreviations

Adm.: Administrative
BMI: Body mass index
CFSWs: Cooks and food service workers
ILO: International Labor Office
MSDs: Musculoskeletal disorders
NMQ: Nordic musculoskeletal disorders questionnaire
OSHA: Occupational Safety and Health Administration
*P*: *P*-value
PPE: Personal protective equipment
SD: Standard deviation
WHO: World Health Organization
WRMSDs: Work-related musculoskeletal disorders
